# TRIM22 suppresses Zika virus replication by targeting NS1 and NS3 for proteasomal degradation

**DOI:** 10.1186/s13578-022-00872-w

**Published:** 2022-08-30

**Authors:** Shulong Zu, Chunfeng Li, Lili Li, Yong-Qiang Deng, Xiang Chen, Dan Luo, Qing Ye, Yi-Jiao Huang, Xiao-Feng Li, Rong-Rong Zhang, Nina Sun, Xianqi Zhang, Saba R. Aliyari, Karin Nielsen-Saines, Jae U. Jung, Heng Yang, Cheng-Feng Qin, Genhong Cheng

**Affiliations:** 1 Center for Systems Medicine, Institute of Basic Medical Sciences, Chinese Academy of Medical Sciences and Peking Union Medical College, Beijing, 100005 China; 2 Institute of Systems Medicine, Chinese Academy of Medical Sciences and Peking Union Medical College, Beijing, 100005 China; 3 Suzhou Institute of Systems Medicine, Suzhou, 215123 Jiangsu China; 4 State Key Laboratory of Pathogen and Biosecurity, Beijing Institute of Microbiology and Epidemiology, Academy of Military Medical Sciences, Beijing, 100071 China; 5 Institute for Immunity, Transplantation and Infection, Department of Pathology, Department of Microbiology and Immunology, Stanford University, Stanford, CA 94305 USA; 6 Department of Microbiology, Immunology and Molecular Genetics, University of California, Los Angeles, CA 90095 USA; 7 Division of Pediatric Infectious Diseases, David Geffen School of Medicine, University of California, Los Angeles, CA 90095 USA; 8 Department of Cancer Biology and Global Center for Pathogens Research and Human Health, Lerner Research Institute, Cleveland Clinic, Cleveland, OH 44195 USA

**Keywords:** TRIM22, Zika virus, Infection, Nonstructural protein, Ubiquitination

## Abstract

**Background:**

Recognition of viral invasion by innate antiviral immune system triggers activation of the type I interferon (IFN-I) and proinflammatory signaling pathways. Subsequently, IFN-I induction regulates expression of a group of genes known as IFN-I-stimulated genes (ISGs) to block viral infection. The tripartite motif containing 22 (TRIM22) is an ISG with strong antiviral functions.

**Results:**

Here we have shown that the TRIM22 has been strongly upregulated both transcriptionally and translationally upon Zika virus (ZIKV) infection. ZIKV infection is associated with a wide range of clinical manifestations in human from mild to severe symptoms including abnormal fetal brain development. We found that the antiviral function of TRIM22 plays a crucial role in counterattacking ZIKV infection. Overexpression of TRIM22 protein inhibited ZIKV growth whereas deletion of *TRIM22* in host cells increased ZIKV infectivity. Mechanistically, TRIM22, as a functional E3 ubiquitin ligase, promoted the ubiquitination and degradation of ZIKV nonstructural protein 1 (NS1) and nonstructural protein 3 (NS3). Further studies showed that the SPRY domain and Ring domain of TRIM22 played important roles in protein interaction and degradation, respectively. In addition, we found that TRIM22 also inhibited other flaviviruses infection including dengue virus (DENV) and yellow fever virus (YFV).

**Conclusion:**

Thus, *TRIM22* is an ISG with important role in host defense against flaviviruses through binding and degradation of the NS1 and NS3 proteins.

**Supplementary Information:**

The online version contains supplementary material available at 10.1186/s13578-022-00872-w.

## Background

Zika virus (ZIKV), dengue virus (DENV), yellow fever virus (YFV), Japanese encephalitis virus (JEV) and West Nile virus (WNV) belong to the genus *Flavivirus* of the family *Flaviviridae*, which infect millions of human annually [1]. In particularly, the newly emerging ZIKV was reported to cause a large outbreak in 2015 in Brazil followed by widespread dissemination across the Americas [2]. ZIKV infection in human was reported in Gujarat and Tamil Nadu states, India, during the year 2016 and 2017 respectively [3]. On October 16, 2018, the Government of India-Ministry of Health and Family Welfare (MoHFW) reported at least 80 positive laboratory confirmed cases of ZIKV infections in Jaipur, Rajasthan, India. Until October 25, 2018, out of 130 positive patients, 32 were pregnant women [4]. Therefore, ZIKV is still a threat to public health and calls for global attentions.

ZIKV contains a single-stranded positive RNA genome encoded into a single polyprotein. The polyprotein is cleaved into the structural proteins, capsid (C), pre-membrane protein (prM) and envelope (E), the nonstructural proteins (NS) including NS1, NS2A, NS2B, NS3, NS4A, NS4B and NS5 [5]. The protein C forms the viral capsid, while prM and E, the main immunogenic glycoproteins of ZIKV, are located on the viral envelope. The NS proteins are involved in replication, assembly of the virus, and/or in antagonizing the host innate immune response, which have been a focus of intensive researches [6]. Among the NS proteins, NS1 modulates the host antibody response and may contribute to viral immune evasion [7, 8]. The function of three transmembrane proteins NS2A, NS4A, and NS4B remains largely elusive, but they are believed to function as scaffold proteins in the viral replication complex. Recently, it was reported that NS2A could promote lysosomal degradation of adherent junction proteins, and NS4A and NS4B can deregulate Akt-mTOR signaling to inhibit neurogenesis [9]. Shah et al. found that NS4A could also interact with ankyrin repeat and LEM domain containing 2 (ANKLE2) to enhance pathogenesis of ZIKV in *Drosophila* [10]. In addition, NS3 is a viral protease which requires NS2B as a cofactor to cleave the viral polyprotein. NS3 also participates in the formation of the viral replication complex, as it contains a helicase, a hydrolase and an RNA-triphosphatase domain [11–15]. Finally, NS5, the largest member of NS proteins, consists of a methyltransferase (MTase) and RNA-dependent RNA polymerase (RdRp) domains. The MTase domain is believed to catalyze both the N7 and 29-O methylation steps, and the RdRp domain synthesizes the viral RNA genome [7]. It was found that NS5 could antagonize IFN-I response by targeting human STAT2 [16].

Innate immune response is the first line of host defense against viral infection, IFNs and ISGs are key components of antiviral innate immunity. Hundreds of ISGs are differentially regulated upon IFN-I induction following infection with a broad range of viruses, such influenza A virus (IAV) and hepatitis C virus (HCV) [17, 18].However, only a subset of ISGs exert antiviral activity against a particular virus. While there are a few ISGs with a strong antiviral activity against a broad spectrum of enveloped viruses including cholesterol-25-hydroxylase (CH25H). CH25H and its enzymatic product, 25-hydroxycholesterol, are critical mediators of host protection against ZIKV infection and its associated microcephaly in a mouse model [19]. Mice deficient in the IFN-I receptor are more susceptible to ZIKV infection in comparison to wide type (WT) mice which highlights the importance of the innate antiviral response and ISGs expression in controlling ZIKV infection [20–22].

TRIM family includes greater than 100 members in humans, some of which had been shown to be ISGs and carry antiviral functions [23, 24]. TRIM22 is a typical TRIM family protein that restricts the replication of various viruses via distinct manners, for example, inhibiting production of human immunodeficiency virus (HIV) progeny [25], suppressing the activity of hepatitis B virus (HBV) core promoter [26] and targeting IAV nucleoprotein for degradation [27]. In this study, we have demonstrated that TRIM22 induces proteasome-dependent degradation of ZIKV NS1 and NS3 proteins as an antiviral mechanism, which could be extended to other flavivirus, such as DENV or YFV.

## Results

### TRIM22 is induced by IFN-α treatment or ZIKV infection

**Fig. 1:**
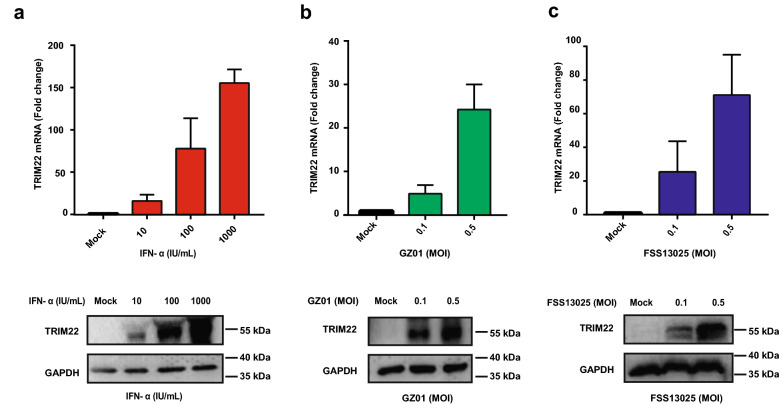
TRIM22 is induced by IFN-α stimulation and ZIKV infection in A549 cells. **a**-**c** qRT-PCR analysis of TRIM22 mRNA and immunoblot analysis of TRIM22 protein in A549 cells stimulated with increasing dose of IFN-α (**a**), two ZIKV strains, GZ01 (**b**) and FSS13025 (**c**). All the cells were harvested at 24 h post treatment

To confirm *TRIM22* as an ISG, we first evaluated that whether TRIM22 was upregulated by IFN-I treatment in A549 cells. We found that both mRNA and protein expression levels of TRIM22 were induced by IFN-α in a dose-dependent manner (10, 100, 100 IU/mL) at 24 h post treatment (Fig. 1a). Then we tested mRNA and protein of TRIM22 in A549 cells infected with either GZ01 or FSS13025 strain of ZIKV. The results showed that infection of these ZIKV strains also induced mRNA and protein expression of TRIM22 (Fig. 1b, c), which is consistent with other ISGs, such as *MX1, IDO1 and RSAD2* (Additional file 1: Fig. S1a–c). These results suggest that *TRIM22* is an ISG induced in response to ZIKV infection.

### TRIM22 inhibits ZIKV replication in vitro

**Fig. 2:**
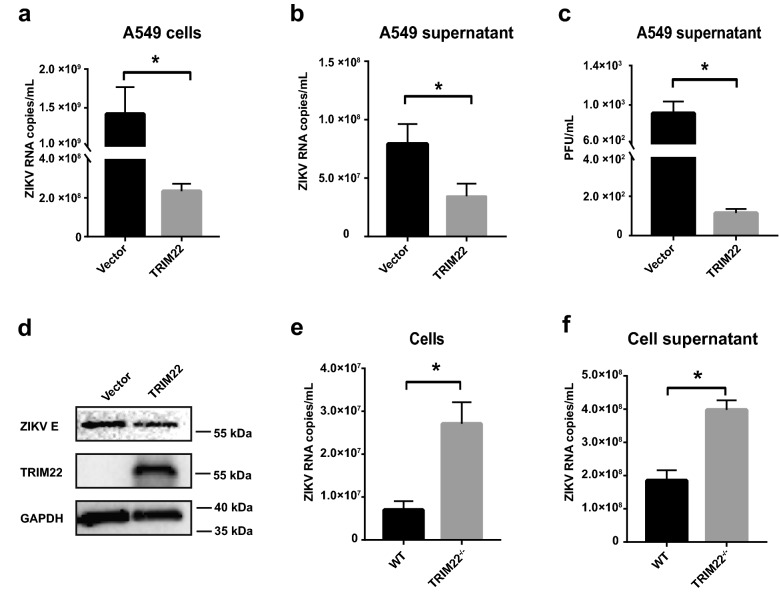
TRIM22 inhibits ZIKV infection in vitro. **a**–**c** TRIM22 was over expressed in A549 cells, the cells were subsequently infected with ZIKV at MOI = 0.1, 12 h post transfection, ZIKV load in cells lysates (**a**) and culture supernatants (**b**) were measured by qRT-PCR or plaque assay (**c**). **d** Western blot analysis of TRIM22 overexpressed A549 cells which were infected with ZIKV. **e**, **f** Wild type and *TRIM22* gene knockout A549 cells were infected with ZIKV at MOI = 0.01. qRT-PCR was performed to detect the ZIKV RNA in cell lysates (**e**) and culture supernatant (**f**). Plaque assay and qRT-PCR data (**a**–**c**, **e** and **f**) are means ± SEM from three independent experiments. **P < 0.05, **P < 0.01 and ***P < 0.001 by Student’s t test

We next determined whether TRIM22 could display anti-ZIKV activity in vitro. A549 cells were transfected with the plasmid overexpressing TRIM22 or an empty vector as control and were subsequently infected with ZIKV (GZ01 strain) at multiplicity of infection (MOI) of 0.1 at 12 h post transfection. The quantitative real-time PCR (qRT-PCR) experiments were performed to detect ZIKV RNA copies in cell lysates (Fig. 2a) and culture supernatants (Fig. 2b) at 48 h post infection (hpi). Culture supernatants of ZIKV infected A549 cells were also tested by plaque assay (Fig. 2c). Further, we also tested the antiviral activity of TRIM22 on neuroblastoma SH-SY5Y cells (Additional file 1: Fig. S2c, d). Western blot and immunofluorescence assay (IFA) were conducted to confirm the inhibitory ability of TRIM22 to ZIKV infection (Fig. 2d and Additional file 1: Fig. S2a). We found that both ZIKV RNA level and protein expression were suppressed in TRIM22 transfected cells, suggesting that overexpression of TRIM22 significantly inhibited the replication of ZIKV in vitro.

To evaluate the effect of endogenous TRIM22 expression on ZIKV infection in vitro, we generated *TRIM22*-knockout A549 (*TRIM22*^*−/−*^ A549) cells by CRISPR/Cas9 system, which was identified by immunoblotting (Additional file 1: Fig. S2b). WT cells and *TRIM22*^*−/−*^ A549 cells were infected with ZIKV at MOI of 0.01, then qRT-PCR experiments were used to test ZIKV RNA copies in cell lysates and culture supernatants (Fig. 2e, f). The result showed more robust ZIKV replication in *TRIM22*^*−/−*^ cells as compared with the WT cells, which is consistent with the overexpression experiments. We also used small interference RNA of TRIM22 in SH-SY5Y cells and the results are consistent with those observed in A549 cells (Additional file 1: Fig. S2e, f). Thus, our studies demonstrated the important role of *TRIM22* as an anti-viral ISG in suppressing ZIKV replication in vitro.

### TRIM22 interacts with ZIKV NS1 and NS3 proteins

**Fig. 3:**
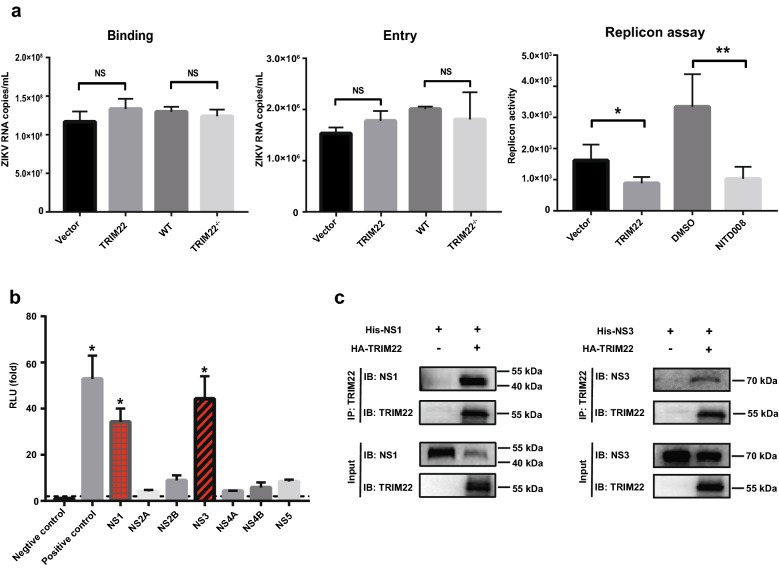
TRIM22 interacts with NS1 and NS3 protein. **a** Measurement of binding, entry and replicon efficiency in TRIM22-overexpressing, and *TRIM22*^*−/−*^ A549 cells. **b** Interactions between TRIM22 and ZIKV nonstructural proteins were screened by a BiLC-based method. Each column represents relative luminescence units from HEK293T cells expressing of individual pair of GlucC-ZIKV and GlucN-TRIM22 compared to controls, detected with Microplate System at 24 h post transfection. **c** Whole-cell lysis of HEK293T cells co-transfected with pHA-TRIM22 and pHis-NS1 (left) or pHis-NS3 (right) expressing plasmids were collected for IP with indicated antibody, followed by western blot detection. Binding, entry and replicon assays data (**a**) are means ± SEM from three independent experiments. **P < 0.05, **P < 0.01 and ***P < 0.001 by Student’s t test

To elucidate which step of ZIKV life cycle is inhibited by TRIM22, the viral binding, entry and replication assays were performed. The results of binding and entry assays showed that TRIM22 did not affect the early step of ZIKV infection (Fig. 3a, left and middle panel). Next, the sub-genomic replicon system which encodes seven ZIKV NS proteins with a Renilla luciferase (RLuc) reporter was used to measure viral replication activity, the result showed that overexpression of TRIM22 reduced the replication activity of the ZIKV RNA genome (Fig. 3a, right panel). Because NS proteins are important to viral replication, we next investigate whether TRIM22 could interact with any ZIKV NS proteins. Bimolecular fluorescence complementation (BiLC) assay was used to investigate protein–protein interaction [28]. TRIM22 was fused with N terminal domain of *Gausssia* luciferase (GluN) onto a lentiviral vector using gateway recombination system, whereas ZIKV NS proteins (NS1, NS2A, NS2B, NS3, NS4A, NS4B and NS5) were connected to C terminal domain of *Gaussia* luciferase (GluC) to produce Glu-C-ZIKV fusion proteins (Additional file 1: Fig. S3a). In this study, we found that TRIM22 specifically interacted with NS1 and NS3, which were also validated by co-immunoprecipitation (Co-IP) experiment (Fig. 3b, c). To further test the relationship between TRIM22 and NS1 or NS3, we performed immunofluorescence staining assay and found that TRIM22 co-localized with NS1 and NS3 (Additional file 1: Fig. S3b). Collectively, these results suggest that TRIM22 interacts with ZIKV NS1 and NS3 proteins.

### TRIM22 degrades NS1 and NS3 proteins through proteasomal degradation

**Fig. 4:**
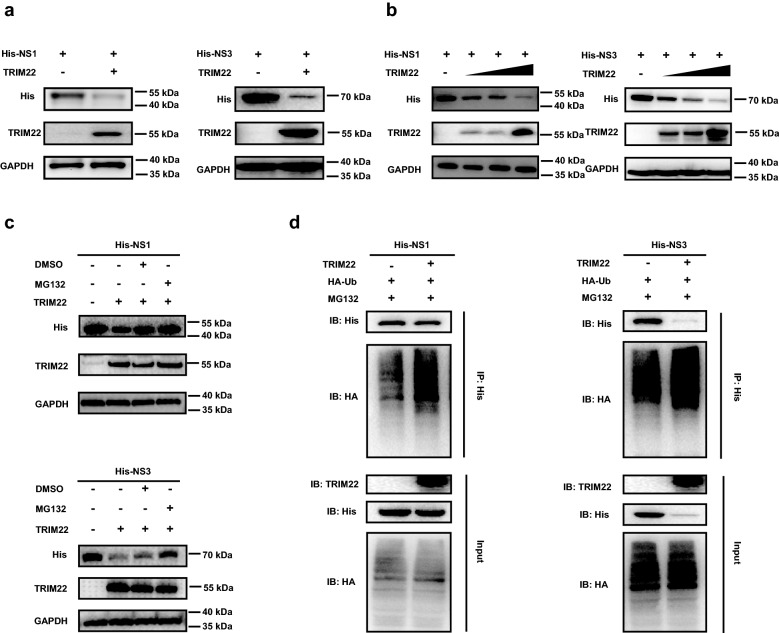
TRIM22 degrade NS1 and NS3 protein by proteasomal degradation. **a** Western blot analysis of lysates from HEK293T cells co-transfected with pHis-NS1 (left) or pHis-NS3 (right) and pM01-TRIM22 or vector control. **b** Western blot analysis of the effect of TRIM22 on degradation of NS1 (left) and NS3 (right) proteins. HEK293T cells were transfected with the pHis-NS1 or pHis-NS3 plasmids and an increasing amount of TRIM22 plasmids (0 ng, 250 ng, 500 ng or 1000 ng, wedges) for 24 h, cells were collected and lysates were probed as indicated. **c** Western blot analysis of lysates from HEK293T cells co-transfected with pM01-TRIM22 and pHis-NS1 (upper) or pHis-NS3 (lower). After 18 h, cells were treated with 10 μM MG132 for 6 h, as indicated. **d** Western blot analysis of lysates IP from HEK293T cells co-transfected with pHA-ubiquitin, pM01-TRIM22 and pHis-NS1 (left) or pHis-NS3 (right) plasmids and treated with MG132, as indicated

To identify the effect of TRIM22 interaction with ZIKV NS1 and NS3 proteins, firstly we investigated that whether ZIKV NS1 and NS3 protein expression was affected in the presence of TRIM22. For this purpose, we transfected HEK293T cells with the plasmids expressing TRIM22 and NS1 or NS3, western blot results showed that TRIM22 overexpression reduced the protein levels of NS1 and NS3 (Fig. 4a) in dose-dependent manner (0, 250, 500 and 1000 ng) (Fig. 4b). TRIM22 family members feature a common E3 ubiquitin ligase domain, thus TRIM22 may be a ubiquitin ligase for NS1 and NS3. To determine whether TRIM22 reduced NS1 and NS3 protein expression through proteasomal degradation, HEK293T cells co-expressing TRIM22 and NS1 or NS3 were treated with the proteasome inhibitor MG132. The results showed that MG132 treatment reduced the TRIM22-dependent degradation of NS1 and NS3 (Fig. 4c), suggesting that TRIM22 mediated NS1 and NS3 degradation through the proteasome pathway. Moreover, we evaluated whether TRIM22 controlled ubiquitylation of NS1 and NS3. HEK293T cells were co-transfected with the plasmids encoding TRIM22, ubiquitin and either NS1 or NS3. Indeed, we observed that TRIM22 overexpression increased the ubiquitylation of NS1 and NS3 (Fig. 4d), which was through K48 but not K63 linkage (Additional file 1: Fig. S4). In summary, these experiments demonstrate that TRIM22 promotes degradation of ZIKV NS1 and NS3 proteins through the ubiquitin–proteasome pathway.

### The Ring domain and SPRY domain of TRIM22 are required for degradation of and interaction with NS1 and NS3 protein, respectively

**Fig. 5:**
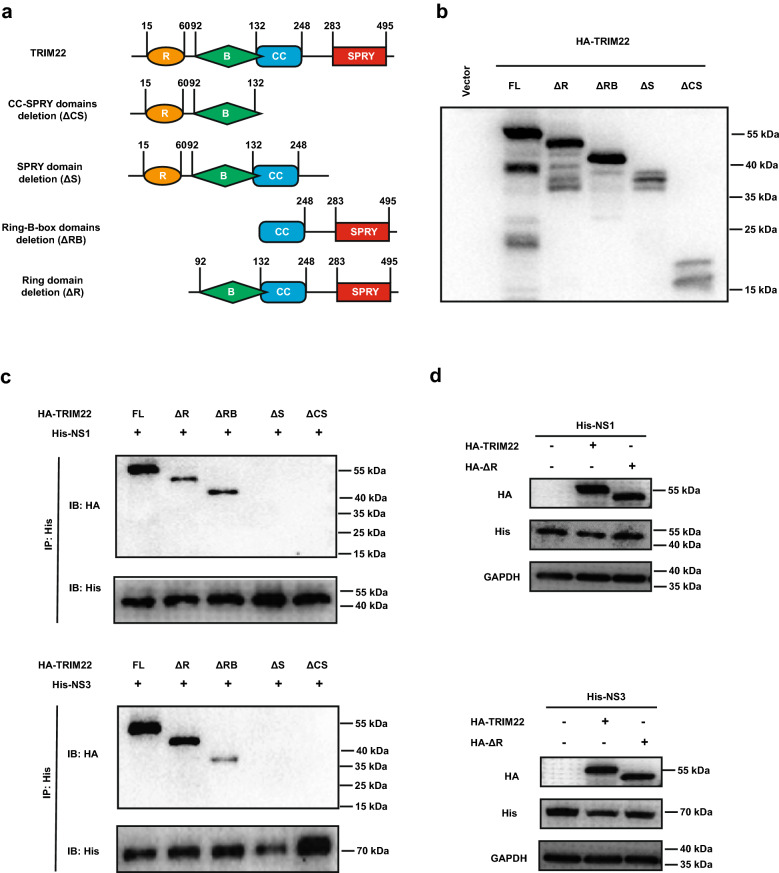
The SPRY domain and Ring domain of TRIM22 are required to interact and degrade with NS1 or NS3 protein, respectively. **a** Schematic diagram of the full length and four truncated forms of TRIM22. **b** Expression of the four TRIM22 truncations were determined by western blotting. **c** Whole-cell lysis of HEK293T cells co-transfected with pHis-NS1(upper) or NS3 (lower) and the indicated four truncated forms of TRIM22 were collected for IP with His tag antibody, followed by western blot analysis. **d** Western blot analysis of lysates from HEK293T cells co-transfected with pHis-NS1 (upper) or pHis-NS3 (lower) and pHA-TRIM22, pHA-TRIM22-Ring-deletion or vector control

We generated four TRIM22 truncations (Fig. 5a) which were tested by western blot (Fig. 5b) to identify which domain is required for the interaction between TRIM22 and NS1 or NS3. We transfected the plasmids expressing TRIM22 truncations and NS1 or NS3 into HEK293T cells. The Co-IP result showed that the SPRY domain of TRIM22 was responsible for the interaction between TRIM22 and NS1 or NS3 (Fig. 5c). The Ring domain of TRIM22 contains a specialized zinc finger and functions as a ubiquitin protein ligase, either alone or as a part of a multi-subunit E3 protein complex [29]. Therefore, we tested that whether the Ring domain deletion of TRIM22 (∆R) would affect NS1 and NS3 degradation. The plasmids encoding TRIM22 or ∆R were co-transfected with NS1 or NS3 into HEK293T cells, and western bolt analysis showed that overexpression of WT but not ∆R reduced NS1 and NS3 protein levels (Fig. 5d). These results suggest that the SPRY domain of TRIM22 interacts with NS1 and NS3, while the Ring domain is essential for TRIM22 to degrade NS1 or NS3 and thus inhibit ZIKV replication.

### TRIM22 inhibits DENV and YFV in vitro

**Fig. 6:**
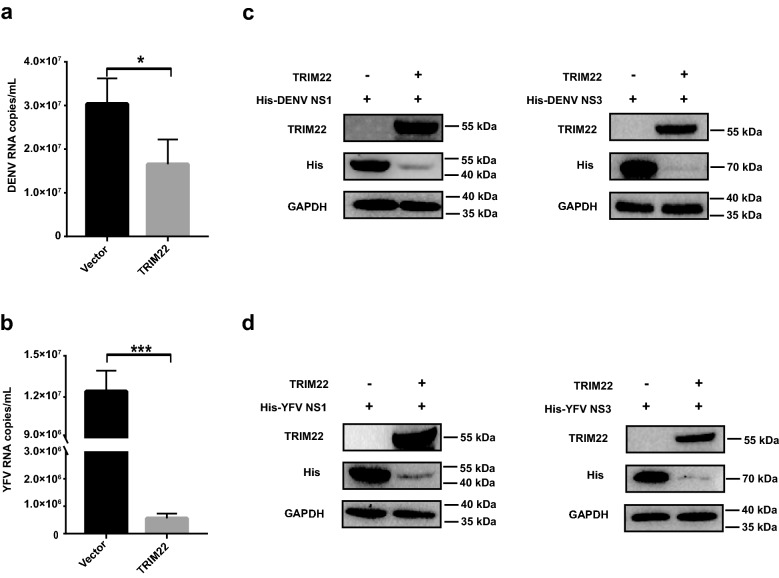
TRIM22 inhibits DENV and YFV infection in vitro. **a**, **b** A549 cells were transfected with TRIM22 expression plasmid or vector control plasmid, 12 h later, the cells were subsequently infected with DENV or YFV at MOI = 0.01. DENV (**a**) and YFV (**b**) in A549 cells lysates were measured by qRT-PCR at 24 hpi. **c**, **d** Western blot analysis of lysates from HEK293T cells co-transfected with pHis-DENV NS1 and NS3 (**c**) or pHis-YFV NS1 and NS3 (**d**), and pM01-TRIM22 or vector control. qRT-PCR data (**a**, **b**) are means ± SEM from three independent experiments. **P < 0.05, **P < 0.01 and ***P < 0.001 by Student’s t test

Since other flaviviruses share similar NS1 and NS3 structures, we expanded the antiviral range of TRIM22 to another two flaviviruses, DENV and YFV. With the similar overexpression experiments, we showed by qRT-PCR analysis that TRIM22 could also suppress DENV and YFV replication in A549 cells (Fig. 6a, b and Additional file 1: Fig. S5a). Furthermore, YFV replicated more robustly in *TRIM22*^*−/−*^ A549 cells than the parental A549 cells (Additional file 1: Fig. S5b), which is consistent with ZIKV. Secondly, we evaluated whether the protein levels of NS1 and NS3 of DENV and YFV were affected by TRIM22. We found that TRIM22 could also degrade the NS1 and NS3 proteins of both DENV (Fig. 6c) and YFV (Fig. 6d). Therefore, our studies suggest that the antiviral activity of TIM22 is not only restricted to ZIKV, but expands to other flaviviruses including DENV and YFV.

## Discussion

The antiviral action of IFN plays a central role in the innate immune response. It stimulates expression of hundreds of ISGs. However, different ISGs may be responsible to restrict infections of different types of viruses [17, 18]. In the present study, we have presented that ZIKV infection, as well as IFN-α treatment, could upregulate endogenous TRIM22 in A549 lung epithelial cells transcriptionally and translationally. Although the antiviral activity of TRIM22 against HIV, IAV, HCV and HBV have been previously reported [25–27, 29], our study extends it to ZIKV, DENV and YFV. We showed that TRIM22 overexpression in A549 cells could inhibit ZIKV infection, whereas *TRIM22*-deficient A549 cells showed enhanced ZIKV replication, implicating *TRIM22* as an anti-ZIKV ISG.

Recently, Chiramel et al*.* found that TRIM5α could restrict tick-borne but not mosquito-borne flaviviruses through proteasomal degradation pathway [30]. Furthermore, they also found that TRIM22 could not impact tick-borne flaviviruses, but have not tested the effects of TRIM22 on mosquito-borne flaviviruses such as ZIKV, DENV and YFV. *TRIM5* and *TRIM22* genes are located adjacent to each on chromosome 11 [31]. Single nucleotide polymorphisms (SNPs) on *TRIM5* and *TRIM22* genes have been implicated to play an important role in several infections disease including HIV [32, 33], HBV [34], measles and rubella vaccination [35, 36]. It is possible that TRIM22 and TRIM5 have acquired different specificities in flavivirus restriction with TRIM22 and TRIM5 to inhibit mosquito-borne and tick-borne flaviviruses, respectively. Future studies are required to further determine the mechanisms responsible for these host restriction specificities.

TRIM22 contains C terminal SPRY domain which is responsible primarily for interaction with target proteins and subcellular localization [23, 37], and N terminal Ring domain which contains a specialized zinc finger structure and serves as a ubiquitin protein ligase, either alone or as a part of a multi-subunit E3 protein complex [29]. Here, we found that WT but not the mutant TRIM22 which lacks the Ring domain could degrade ZIKV NS1 and NS3 proteins. This suggest that the Ring domain is critical for TRIM22 mediated ubiquitination and degradation of ZIKV NS1 and NS3 proteins. Interestingly we also found that TRIM22 lacking the SPRY domain failed to interact with ZIKV NS1 and NS3 proteins, suggesting that the SPRY domain is important for its binding to viral targets. The antiviral properties of TRIM22 by ubiquitination have been previously reported, for instance, TRIM22 could inhibit HCV by degrading NS5A protein, while suppressing IAV by targeting nucleoprotein [27, 29]. In this study, we found that TRIM22 could significantly inhibit ZIKV replication in vitro. Importantly, our results showed that TRIM22 could significantly down-regulate the expression of NS1 and NS3 proteins, which are critical for the viral replication and immune evasion.

In summary, our findings indicate that TRIM22 could inhibit ZIKV replication through its SPRY domain to interact with NS1 and NS3 and its Ring domain to degrade the NS1 and NS3 proteins by ubiquitination. In addition, we also find that TRIM22 could inhibit other flavivirus, such as DENV and YFV in vitro. Taken together, this work expands the antiviral function of TRIM22 and illuminates the mechanism underlying TRIM22-mediated inhibition of ZIKV replication, which provides molecular basis for the potential development of novel anti-ZIKV therapeutics.

## Methods

### Virus, cells and reagents

ZIKV strains (GZ01, GenBank: KU820898, FSS13025/2010, GenBank: JN860885), DENV-2 (43 strain, GenBank: AF204178) and YFV 17D were described in our previous work [19, 38]. A549, BHK-21, Vero and HEK293T cells were purchased from American Type Culture Collection (ATCC) and cultured in Dulbecco’s modified Eagle’s medium (DMEM), supplied with 10% fetal bovine serum (FBS), penicillin (100 Unit/mL) and streptomycin (100 μg/mL). SH-SY5Y cell was purchased from ATCC and cultured in DMEM/F12, supplied with 10% fetal bovine serum (FBS), penicillin (100 Unit/mL) and streptomycin (100 μg/mL). All cells were grown at 37 °C in 5% CO_2_. MG132 was purchased from Sigma-Aldrich (M8699), and NITD008, an adenosine nucleoside analog inhibitor that inhibits the RdRp (RNA-dependent RNA polymerase) activity of flavivirus [39, 40], was a gift from P.-Y. Shi (The University of Texas Medical Branch). Mouse anti–GAPDH antibody (COBIO), rabbit anti-TRIM22 antibody (NOVUSBIO), mouse anti-HA antiboy (COBIO) and anti-His tag antibody (CST) were used for detection at the appropriate dilutions.

### Plasmid

pM01-GFP and pM01-TRIM22 (Homo sapiens) expression plasmids were purchased from GeneCopoeia company. His tagged ZIKV NS1 and NS3 expression plasmids, pcDNA6/V5-His-NS1 (pHis-NS1) and pcDNA6/V5-His-NS3 (pHis-NS3), were previously constructed and preserved by our laboratory [41]. pcDNA6/V5-His-NS1/NS3(DENV) and pcDNA6/V5-His-NS1/NS3(YFV) were constructed by the same techniques as before. pHA-TRIM22 and TRIM22 truncations were cloned using standard molecular cloning. pHA-ubiquitin, pHA-K48, pHA-K63 plasmids and BiLC lentiviral vectors were provided by Dr. Xiao-Feng Qin (CAMS) [28, 42].

### Plaque assay

BHK-21 cells were seeded in a 12-well plate for 12 h. Cells were washed with PBS once and infected with serially diluted virus samples for 1 h. Then the cells were treated with medium containing low melting temperature agarose (1%) and FBS (2%) after supernatants were removed. 4 days post-infection, cells were fixed using paraformaldehyde [4% (wt/vol)] and were stained with crystal violet (1%). Plaques were counted and multiplied by the dilution factor to determine the plaque forming unit (PFU).

### ZIKV binding, entry, and replicon assay

ZIKV cell binding and entry experiments were performed on the basis of the protocol described previously [43]. For binding analysis, vector, TRIM22-overexpressing, WT and *TRIM22*^*−/−*^ A549 cells were incubated with ZIKV at 4 °C for 1 h. Then the cells were washed with PBS. The inhibition of TRIM22 to the binding of ZIKV and cell surface was assessed by measuring the viral copy number in the cell lysates by qRT-PCR; Entry: vector, TRIM22-overexpressing, WT and *TRIM22*^*−/−*^ A549 cells were treated as binding analysis, then incubated at 37 °C for another 10 min. Viral entry into cells was assessed by determining the viral copy number in the cell lysates by qRT-PCR; Replicon: the replicon assay of SZ01 ZIKV was performed as previously published[44, 45], with minor modifications. Briefly, BHK-21 cells were seeded in a 24-well plate and then transfected with 200 ng of the in vitro-transcribed replicon containing the seven ZIKV nonstructural proteins and the RLuc reporter and with 200 ng of pM01-TRIM22 or control plasmid using a Lipofectamine 3000 reagent (Thermo Fisher Scientific), NITD008 was used as a positive control. After 48 h, the cell lysates were collected, the RLuc activity was measured using the Renilla Luciferase Assay system (Promega) with a GloMax 96 microplate luminometer.

### Immunoprecipitation and immunoblotting

HEK293T cells were transfected with the indicated plasmids. 30 h after transfection, the total protein of the cells was extracted using Cytobuster Protein Extraction Reagent (Sigma) containing complete EDTA-free Protease Inhibitor Cocktail (Roche). An aliquot of the extracts was immunoblotted with the indicated antibodies. The remaining extracts were immunoprecipitated using Sepharose beads bound to anti-HA or anti-His antibody (Sigma-Aldrich) at 4 °C overnight. The Sepharose beads were washed with wash buffer four times, then proteins were eluted by heating the beads to 98 °C in 1 × SDS–polyacrylamide gel electrophoresis loading buffer (Genstar). The eluate and remaining whole cell extracts were analyzed by immunoblotting with the indicated antibodies. Immunoblotting was carried out by standard procedures as usual.

### Gene knockout by the CRISPR/Cas9 system

To knockout *TRIM22* in the A549 cell line, two small guide RNAs (sgRNAs) (~ 100-base pair gap sequence) targeting the gene were designed and cloned into sgRNA expression vectors under the control of the U6 promoter as described previously [46, 47]. The sequence of sgRNAs of TRIM22 were as followed: sgRNA1: 5′-CACCGGATCGAGAGACAGAAGATTC-3′; sgRNA2: 5′-CACCGGCGGAGGTTGAGGGGATCGT-3′. A549 cells were co-transfected with sgRNAs and Cas9 expression plasmids, followed by puromycin selection. Single clones were isolated by FACS and confirmed by PCR genotyping and sequencing.

### RNA interference

Three small interference RNAs (siRNAs) targeting human TRIM22 and a negative control (NC) siRNA were designed and synthesized by RIBOBIO. SH-SY5Y cells were seeded in a 24-well plate. After 18 h, the cells were transfected with TRIM22 siRNAs at a final concentration of 100 nM. After another 24 h, the cells were collected for immunoblotting or infected with GZ01 at MOI = 0.01 for analyzing virus replication.

### RNA preparation and real-time PCR

Total RNA from cells or cell supernatants were extracted with the PureLink RNA Extraction kit (Thermo Fisher Scientific). Viral RNA copies were measured by qRT-PCR [48] with the One Step PrimeScript RT-PCR kit (Takara). ZIKV primers and TaqMan probes were described previously [49]. SYBR Green qPCR mix (TransGen Biotech, Beijing) was used to quantify the mRNA level of the ISGs. Primers used in this study are listed in Additional file 1: Table S1.

### Immunofluorescence staining

Vero cells were transfected with pHA-TRIM22 and pHis-NS1 or NS3 plasmids for 24 h. The cells were then fixed with 4% paraformaldehyde and permeabilized in 0.2% Triton X-100 at room temperature. The cells were washed with PBS three times. Anti-HA tag and anti-His tag antibody were incubated for 1 h, and goat anti-mouse or rabbit second antibody were incubated for another hour. Nuclei were stained with DAPI (0.5 μg/mL). Finally, the images were obtained by fluorescence microscope.

### Statistical analysis

All data were analyzed using Prism software (GraphPad). Statistical evaluation was performed by two-way Student’s t test. Data are means ± SEM, and P values are indicated by *P < 0.5, **P < 0.01, and ***P < 0.001. For western blot data, representative data from at least two repeats was shown. All cellular experiments were repeated at least three times.

## Supplementary Information

**Additional file 1: Figure S1.** ISGs are induced by ZIKV infection in A549 cells. **a**-**c** qRT-PCR analysis of *MX1*, *IDO1 and RSAD2* mRNA in A549 cells stimulated with increasing dose of ZIKV (FSS13025) infection. **Figure S2.** TRIM22 inhibits ZIKV infection in vitro. **a** Vero cells in 24-well plate were transfected with increasing amount of pM01-TRIM22 plasmid, 12 h later, the cells were infected with ZIKV GZ01 at MOI = 0.01, IFA of ZIKV E protein was conducted at 24 hpi. Scale bar, 100 μm. **b** Western blot analysis of lysates from A549 cells and *TRIM22*^*−/−*^ A549 cells. **c-d** SH-SY5Y cells were transfected with pM01-TRIM22 plasmid, 12 h later, the cells were infected with ZIKV GZ01 at MOI = 0.1, ZIKV loads in cell lysates were measured by qRT-PCR at 24 hpi (**c**) and 48 hpi (**d**). **e** Western blot analysis of lysates from SH-SY5Y cells which were transfected with either NC siRNA or TRIM22 siRNAs. **f** SH-SY5Y cells were transfected with NC siRNA or TIRM22 siRNAs, 24 h later, the cells were infected with ZIKV GZ01 at MOI = 0.01, ZIKV loads in cell lysates were measured by qRT-PCR at 48 hpi. **Figure S3.** Model of BiLC system and IFA used to test the interactions between TRIM22 and ZIKV proteins. **a** Work model of GlucN-TRIM22 and GlucC-ZIKV NS1 or NS3 in BiLC system. **b** IFA of A549 cells that were co-transfected with pHA-TRIM22 and pHis-NS1 or pHis-NS3 plasmids, stained with HA and His tag antibody, then imaged by fluorescence microscope. Scale bar, 10 μm. **Figure S4. TRIM22 mediates the K48 ubiquitylation of ZIKV NS1 and NS3 proteins.** Western blot analysis of total cell lysates or immunoprecipitated proteins from HEK293T cells co-transfected with pM01-TRIM22, pHis-NS1 or pHis-NS3 and pHA-K48 (**a**) or pHA-K63(**b**) plasmids and treated with MG132, as indicated. **Figure S5.** TRIM22 inhibits YFV infection in vitro. **a** TRIM22 was over expressed in A549 cells, the cells were subsequently infected with YFV at MOI = 0.01, 12 h post transfection, YFV load in culture supernatant was measured by qRT-PCR. **b** *TRIM22*^*−/−*^A549 cells were infected with YFV, the cell lysates were measured by qRT-PCR. qRT-PCR data are means ± SEM from three independent experiments. **P < 0.05, **P < 0.01 and ***P < 0.001 by Student’s t test. **Table S1.** The list of ISG, ZIKV, DENV and YFV primers used in this study.

## Data Availability

The datasets used or analyzed during the current study are available from the corresponding author on reasonable request.

## Abbreviations

IFN: Interferon
ISGs: Interferon stimulated genes
TRIM22: Tripartite motif containing 22
ZIKV: Zika virus
DENV: Dengue virus
YFV: Yellow fever virus
NS protein: Nonstructural protein

## References

[1] Heinz FX, Stiasny K (2012). Flaviviruses and flavivirus vaccines. Vaccine.

[2] Petersen LR, Jamieson DJ, Honein MA (2016). Zika virus. N Engl J Med.

[3] Yadav PD, Malhotra B, Sapkal G, Nyayanit DA, Deshpande G, Gupta N (2019). Zika virus outbreak in Rajasthan, India in 2018 was caused by a virus endemic to Asia. Infect Genet Evol.

[4] Saxena SK, Kumar S, Sharma R, Maurya VK, Dandu HR, Bhatt MLB (2019). Zika virus disease in India—update October 2018. Travel Med Infect Dis.

[5] Kuno G, Chang GJ (2007). Full-length sequencing and genomic characterization of Bagaza, Kedougou, and Zika viruses. Arch Virol.

[6] Asif A, Manzoor S, Tuz-Zahra F, Saalim M, Ashraf M, Ishtiyaq J (2017). Zika virus: immune evasion mechanisms, currently available therapeutic regimens, and vaccines. Viral Immunol.

[7] Klema VJ, Padmanabhan R, Choi KH (2015). Flaviviral replication complex: coordination between RNA Synthesis and 5′-RNA capping. Viruses.

[8] Song H, Qi J, Haywood J, Shi Y, Gao GF (2016). Zika virus NS1 structure reveals diversity of electrostatic surfaces among flaviviruses. Nat Struct Mol Biol.

[9] Yoon KJ, Song G, Qian X, Pan J, Xu D, Rho HS (2017). Zika-virus-encoded NS2A disrupts mammalian cortical neurogenesis by degrading adherens junction proteins. Cell Stem Cell.

[10] Liang Q, Luo Z, Zeng J, Chen W, Foo SS, Lee SA (2016). Zika virus NS4A and NS4B proteins deregulate Akt-mTOR signaling in human fetal neural stem cells to inhibit neurogenesis and induce autophagy. Cell Stem Cell.

[11] Tian H, Ji X, Yang X, Zhang Z, Lu Z, Yang K (2016). Structural basis of Zika virus helicase in recognizing its substrates. Protein Cell.

[12] Lei J, Hansen G, Nitsche C, Klein CD, Zhang L, Hilgenfeld R (2016). Crystal structure of Zika virus NS2B-NS3 protease in complex with a boronate inhibitor. Science.

[13] Jain R, Coloma J, Garcia-Sastre A, Aggarwal AK (2016). Structure of the NS3 helicase from Zika virus. Nat Struct Mol Biol.

[14] Zhang Z, Li Y, Loh YR, Phoo WW, Hung AW, Kang C (2016). Crystal structure of unlinked NS2B-NS3 protease from Zika virus. Science.

[15] Phoo WW, Li Y, Zhang Z, Lee MY, Loh YR, Tan YB (2016). Structure of the NS2B-NS3 protease from Zika virus after self-cleavage. Nat Commun.

[16] Grant A, Ponia SS, Tripathi S, Balasubramaniam V, Miorin L, Sourisseau M (2016). Zika virus targets human STAT2 to inhibit type I interferon signaling. Cell Host Microbe.

[17] Schneider WM, Chevillotte MD, Rice CM (2014). Interferon-stimulated genes: a complex web of host defenses. Annu Rev Immunol.

[18] Liu SY, Aliyari R, Chikere K, Li G, Marsden MD, Smith JK (2013). Interferon-inducible cholesterol-25-hydroxylase broadly inhibits viral entry by production of 25-hydroxycholesterol. Immunity.

[19] Li C, Deng YQ, Wang S, Ma F, Aliyari R, Huang XY (2017). 25-Hydroxycholesterol protects host against Zika virus infection and its associated microcephaly in a mouse model. Immunity.

[20] Yockey LJ, Varela L, Rakib T, Khoury-Hanold W, Fink SL, Stutz B (2016). Vaginal exposure to Zika virus during pregnancy leads to fetal brain infection. Cell.

[21] Aliota MT, Caine EA, Walker EC, Larkin KE, Camacho E, Osorio JE (2016). Characterization of lethal Zika virus infection in AG129 mice. PLoS Negl Trop Dis.

[22] Lazear HM, Govero J, Smith AM, Platt DJ, Fernandez E, Miner JJ (2016). A mouse model of Zika virus pathogenesis. Cell Host Microbe.

[23] Ozato K, Shin DM, Chang TH, Morse HC (2008). TRIM family proteins and their emerging roles in innate immunity. Nat Rev Immunol.

[24] Versteeg GA, Rajsbaum R, Sanchez-Aparicio MT, Maestre AM, Valdiviezo J, Shi M (2013). The E3-ligase TRIM family of proteins regulates signaling pathways triggered by innate immune pattern-recognition receptors. Immunity.

[25] Barr SD, Smiley JR, Bushman FD (2008). The interferon response inhibits HIV particle production by induction of TRIM22. PLoS Pathog.

[26] Gao B, Duan Z, Xu W, Xiong S (2009). Tripartite motif-containing 22 inhibits the activity of hepatitis B virus core promoter, which is dependent on nuclear-located RING domain. Hepatology.

[27] Di Pietro A, Kajaste-Rudnitski A, Oteiza A, Nicora L, Towers GJ, Mechti N (2013). TRIM22 inhibits influenza A virus infection by targeting the viral nucleoprotein for degradation. J Virol.

[28] Li C, Wang Z, Cao Y, Wang L, Ji J, Chen Z (2017). Screening for novel small-molecule inhibitors targeting the assembly of influenza virus polymerase complex by a bimolecular luminescence complementation-based reporter system. J Virol.

[29] Yang C, Zhao X, Sun D, Yang L, Chong C, Pan Y (2016). Interferon alpha (IFNalpha)-induced TRIM22 interrupts HCV replication by ubiquitinating NS5A. Cell Mol Immunol.

[30] Chiramel AI, Meyerson NR, McNally KL, Broeckel RM, Montoya VR, Mendez-Solis O (2019). TRIM5alpha restricts flavivirus replication by targeting the viral protease for proteasomal degradation. Cell Rep..

[31] Sawyer SL, Emerman M, Malik HS (2007). Discordant evolution of the adjacent antiretroviral genes TRIM22 and TRIM5 in mammals. PLoS Pathog.

[32] Santa-Marta M, de Brito PM, Godinho-Santos A, Goncalves J (2013). Host factors and HIV-1 replication: clinical evidence and potential therapeutic approaches. Front Immunol.

[33] Ghezzi S, Galli L, Kajaste-Rudnitski A, Turrini F, Marelli S, Toniolo D (2013). Identification of TRIM22 single nucleotide polymorphisms associated with loss of inhibition of HIV-1 transcription and advanced HIV-1 disease. AIDS.

[34] Zhao N, Wang XL, Gu QH, Huang F, Zheng W, Li ZW (2014). Tripartite motif-containing 22 gene -364T/C polymorphism associated with hepatitis B virus infection in Chinese Han population. Hepat Mon.

[35] Ovsyannikova IG, Haralambieva IH, Dhiman N, O'Byrne MM, Pankratz VS, Jacobson RM (2010). Polymorphisms in the vitamin A receptor and innate immunity genes influence the antibody response to rubella vaccination. J Infect Dis.

[36] Ovsyannikova IG, Haralambieva IH, Vierkant RA, O'Byrne MM, Poland GA (2013). Associations between polymorphisms in the antiviral TRIM genes and measles vaccine immunity. Hum Immunol.

[37] Hattlmann CJ, Kelly JN, Barr SD (2012). TRIM22: a diverse and dynamic antiviral protein. Mol Biol Int.

[38] Li XF, Dong HL, Wang HJ, Huang XY, Qiu YF, Ji X (2018). Development of a chimeric Zika vaccine using a licensed live-attenuated flavivirus vaccine as backbone. Nat Commun.

[39] Lo MK, Shi PY, Chen YL, Flint M, Spiropoulou CF (2016). In vitro antiviral activity of adenosine analog NITD008 against tick-borne flaviviruses. Antiviral Res.

[40] Deng Y-Q, Zhang N-N, Li C-F, Tian M, Hao J-N, Xie X-P (2016). Adenosine analog NITD008 is a potent inhibitor of Zika virus. Open Forum Infect Dis.

[41] Li L, Zhao H, Liu P, Li C, Quanquin N, Ji X (2018). PARP12 suppresses Zika virus infection through PARP-dependent degradation of NS1 and NS3 viral proteins. Sci Signal.

[42] Wang S, Chen Y, Li C, Wu Y, Guo L, Peng C (2016). TRIM14 inhibits hepatitis C virus infection by SPRY domain-dependent targeted degradation of the viral NS5A protein. Sci Rep.

[43] Zhu X, He Z, Yuan J, Wen W, Huang X, Hu Y (2015). IFITM3-containing exosome as a novel mediator for anti-viral response in dengue virus infection. Cell Microbiol.

[44] Liu ZY, Li XF, Jiang T, Deng YQ, Zhao H, Wang HJ (2013). Novel cis-acting element within the capsid-coding region enhances flavivirus viral-RNA replication by regulating genome cyclization. J Virol.

[45] Xie X, Zou J, Shan C, Yang Y, Kum DB, Dallmeier K (2016). Zika virus replicons for drug discovery. EBioMedicine.

[46] Cong L, Ran FA, Cox D, Lin S, Barretto R, Habib N (2013). Multiplex genome engineering using CRISPR/Cas systems. Science.

[47] Mali P, Yang L, Esvelt KM, Aach J, Guell M, DiCarlo JE (2013). RNA-guided human genome engineering via Cas9. Science.

[48] Johnson BW, Russell BJ, Lanciotti RS (2005). Serotype-specific detection of dengue viruses in a fourplex real-time reverse transcriptase PCR assay. J Clin Microbiol.

[49] Deng YQ, Zhao H, Li XF, Zhang NN, Liu ZY, Jiang T (2016). Isolation, identification and genomic characterization of the Asian lineage Zika virus imported to China. Sci China Life Sci.

