# Supplementary material for: TRIM22 suppresses Zika virus replication by targeting NS1 and NS3 for proteasomal degradation
Source: Cell Biosci. 2022 Aug 30;12:139. doi: 10.1186/s13578-022-00872-w (PMC9429444; doi:10.1186/s13578-022-00872-w)

**Additional file 1**

**TRIM22 suppresses zika virus replication by targeting NS1 and NS3 for proteasomal degradation**

Shulong Zu^1,2,3,4^, Chunfeng Li^5^, Lili Li^2,3^, Yong-Qiang Deng^4^, Xiang Chen^4^, Dan Luo^4^, Qing Ye^4^, Yi-Jiao Huang^4^, Xiao-Feng Li^4^, Rong-Rong Zhang^4^, Nina Sun^1,2,3^, Xianqi Zhang^1^, [Saba R. Aliyari](https://pubmed.ncbi.nlm.nih.gov/?sort=date&term=Aliyari+R&cauthor_id=34514088)^6^, [Karin Nielsen-Saines](https://pubmed.ncbi.nlm.nih.gov/?sort=date&term=Nielsen-Saines+K&cauthor_id=33510473)^7^, [Jae U Jung](https://pubmed.ncbi.nlm.nih.gov/?sort=date&term=Jung+JU&cauthor_id=33510473)^8^, Heng Yang^2,3*^, Cheng-Feng Qin^4*^, Genhong Cheng^6*^

^1^Center for Systems Medicine, Institute of Basic Medical Sciences, Chinese Academy of Medical Sciences and Peking Union Medical College, Beijing 100005, China

^2^ Institute of Systems Medicine, Chinese Academy of Medical Sciences and Peking Union Medical College, Beijing 100005, China.

^3^Suzhou Institute of Systems Medicine, Suzhou, Jiangsu 215123, China.

^4^State Key Laboratory of Pathogen and Biosecurity, Beijing Institute of Microbiology and Epidemiology, Academy of Military Medical Sciences, Beijing 100071, China.

^5^Institute for Immunity, Transplantation and Infection, Department of Pathology, Department of Microbiology and Immunology, Stanford University, Stanford, CA 94305, USA.

^6^Department of Microbiology, Immunology and Molecular Genetics, University of California, Los Angeles, CA 90095, USA.

^7^Division of Pediatric Infectious Diseases, David Geffen School of Medicine, University of California, Los Angeles, Los Angeles, CA 90095, USA.

^8^Department of Cancer Biology and Global Center for Pathogens Research and Human Health, Lerner Research Institute, Cleveland Clinic, Cleveland, OH 44195, USA.

G.C., C-.F.Q. H.Y. and are senior co-authors of this paper.

*Corresponding author. Email: [gcheng@mednet.ucla.edu](mailto:gcheng@mednet.ucla.edu) (G.C.); [qincf@bmi.ac.cn](mailto:qincf@bmi.ac.cn) (C-.F.Q.); [yhmyt@hotmail.com](mailto:yhmyt@hotmail.com) (H.Y.)

**Additional figures**

**Figure S1. ISGs are induced by ZIKV infection in A549 cells. a**-**c** qRT-PCR analysis of *MX1*, *IDO1 and RSAD2* mRNA in A549 cells stimulated with increasing dose of ZIKV (FSS13025) infection.


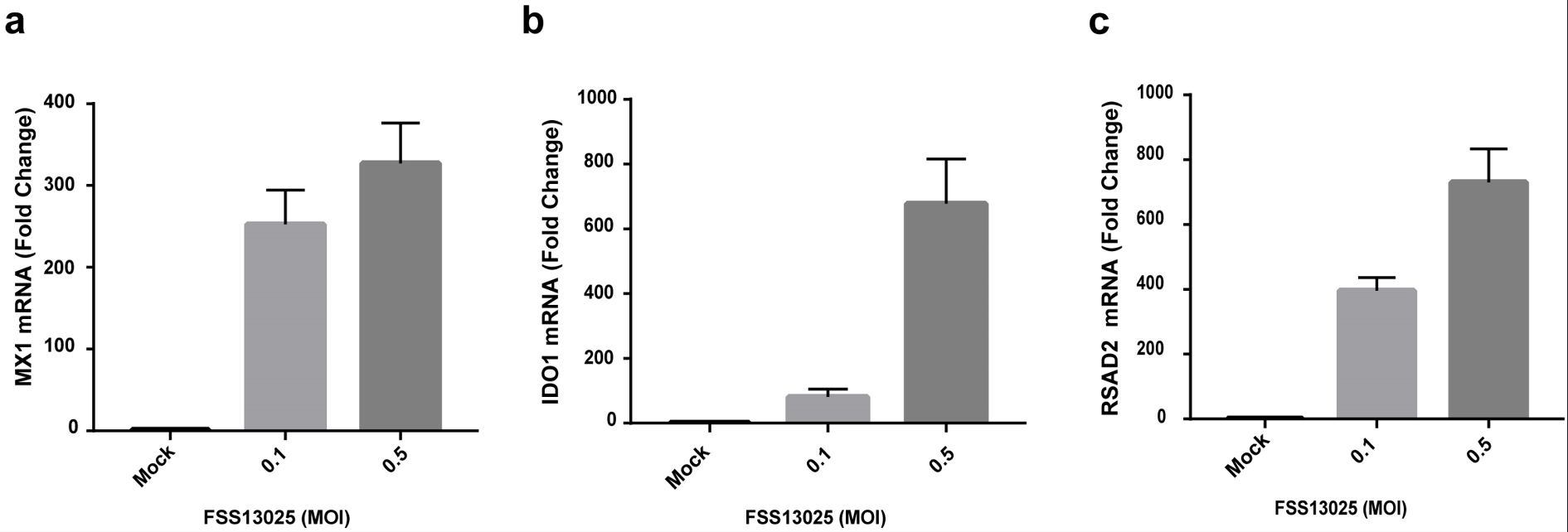


**Figure S2. TRIM22 inhibits ZIKV infection *in vitro*.** **a** Vero cells in 24-well plate were transfected with increasing amount of pM01-TRIM22 plasmid, 12 hours later, the cells were infected with ZIKV GZ01 at MOI=0.01, IFA of ZIKV E protein was conducted at 24 hours post-infection. Scale bar, 100 μm. **b** Western blot analysis of lysates from A549 cells and *TRIM22^-/-^* A549 cells. **c-d** SH-SY5Y cells were transfected with pM01-TRIM22 plasmid, 12 hours later, the cells were infected with ZIKV GZ01 at MOI=0.1, ZIKV loads in cell lysates were measured by qRT-PCR at 24 hpi (**c**) and 48 hpi (**d**). **e** Western blot analysis of lysates from SH-SY5Y cells which were transfected with either negative control (NC) small interfere RNA (siRNA) or TRIM22 siRNAs. **f** SH-SY5Y cells were transfected with NC siRNA and TIRM22 siRNAs, 24 hours later, the cells were infected with ZIKV GZ01 at MOI=0.01, ZIKV loads in cell lysates were measured by qRT-PCR at 48 hpi.


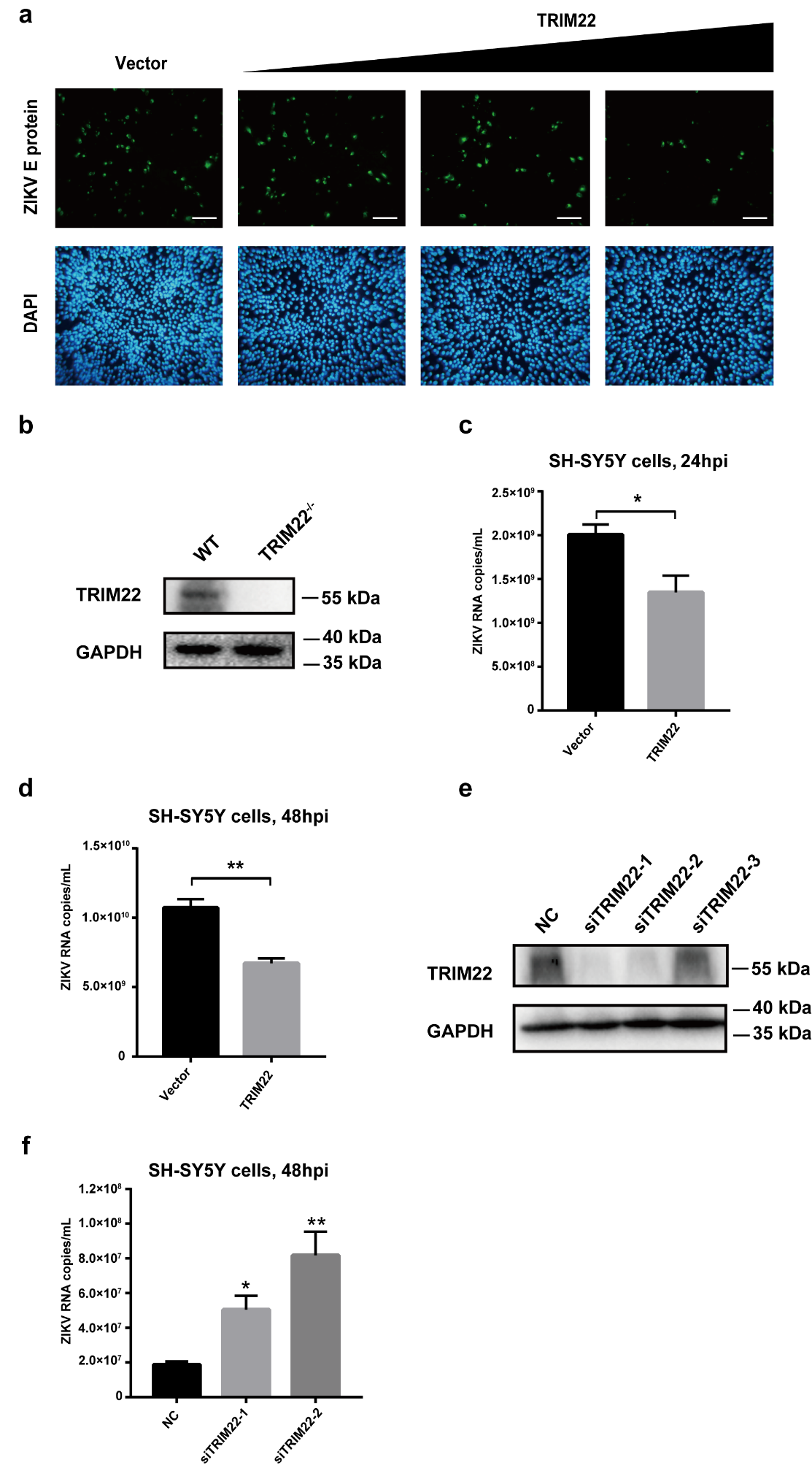


**Figure S3.** **Model of BiLC system and IFA used to test the interactions between TRIM22 and ZIKV proteins. a** Work model of GlucN-TRIM22 and GlucC-ZIKV NS1 or NS3 in BiLC system. **b** IFA of A549 cells that were co-transfected with pHA-TRIM22 and pHis-NS1 or pHis-NS3 plasmids, stained with HA and His tag antibody, then imaged by fluorescence microscope. Scale bar, 10 μm.


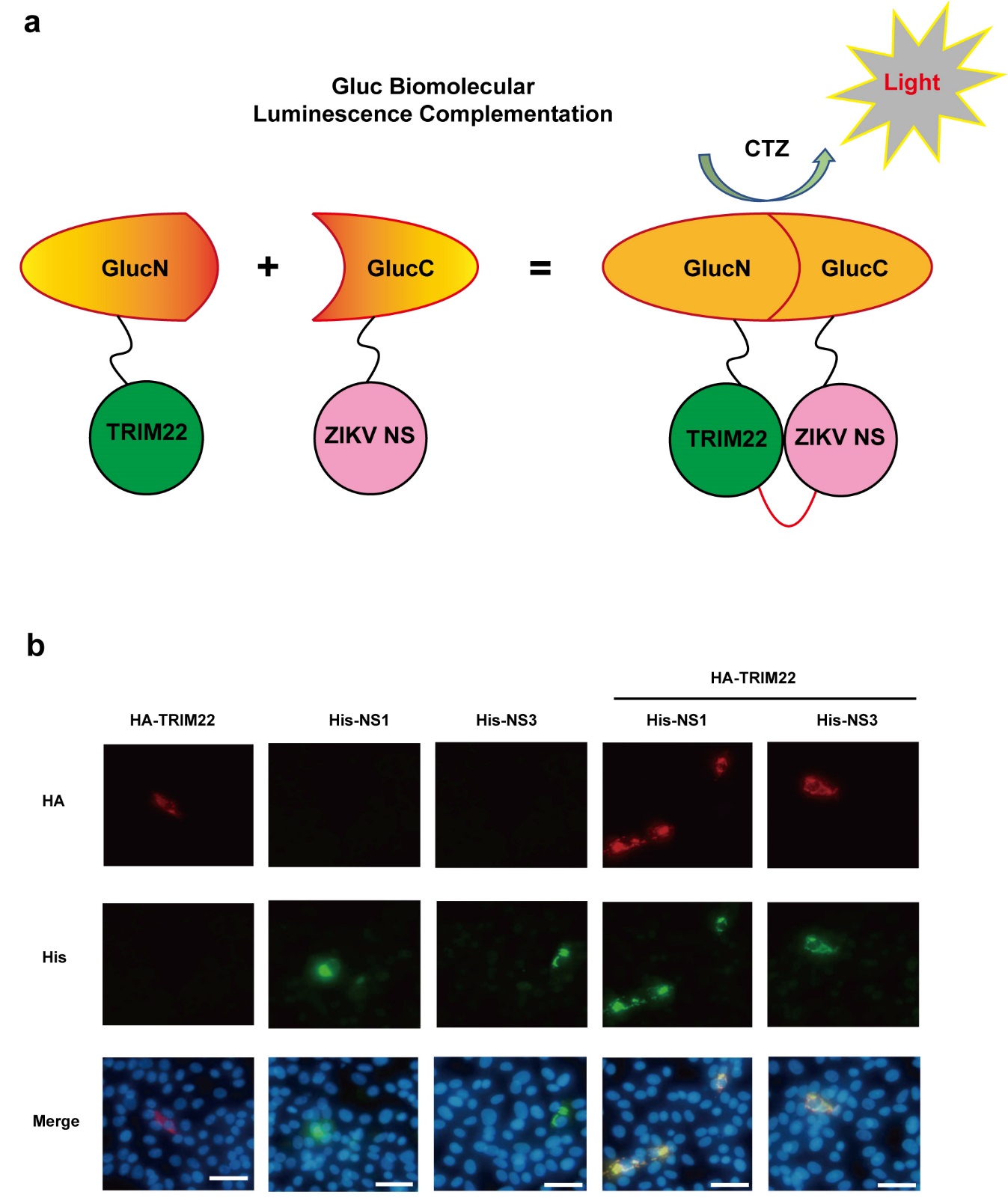


**Figure S4.** **TRIM22 mediates the K48 ubiquitylation of ZIKV NS1 and NS3 proteins.** Western blot analysis of total cell lysates or immunoprecipitated proteins from HEK293T cells co-transfected with pM01-TRIM22, pHis-NS1 or pHis-NS3 and HA-K48 (**a**) or HA-K63(**b**) plasmids and treated with MG132, as indicated.


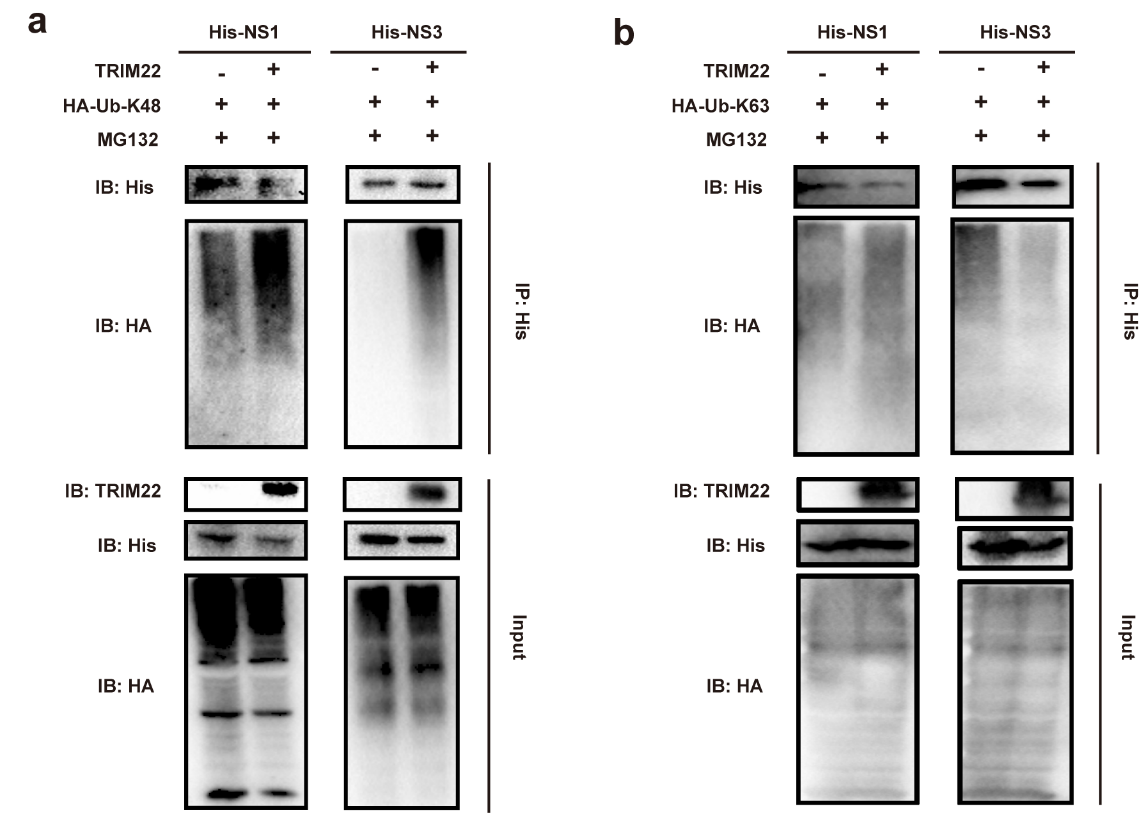


**Figure S5. TRIM22 inhibits YFV infection *in vitro*. a** TRIM22 was over expressed in A549 cells, the cells were subsequently infected with YFV at MOI=0.01, 12 hours post transfection, YFV load in culture supernatant was measured by qRT-PCR. **b** *TRIM22^-/-^*A549 cells were infected with YFV, the cell lysates were measured by qRT-PCR. qRT-PCR data are means ± SEM from three independent experiments. **P < 0.05, **P < 0.01 and ***P < 0.001 by Student’s t test.


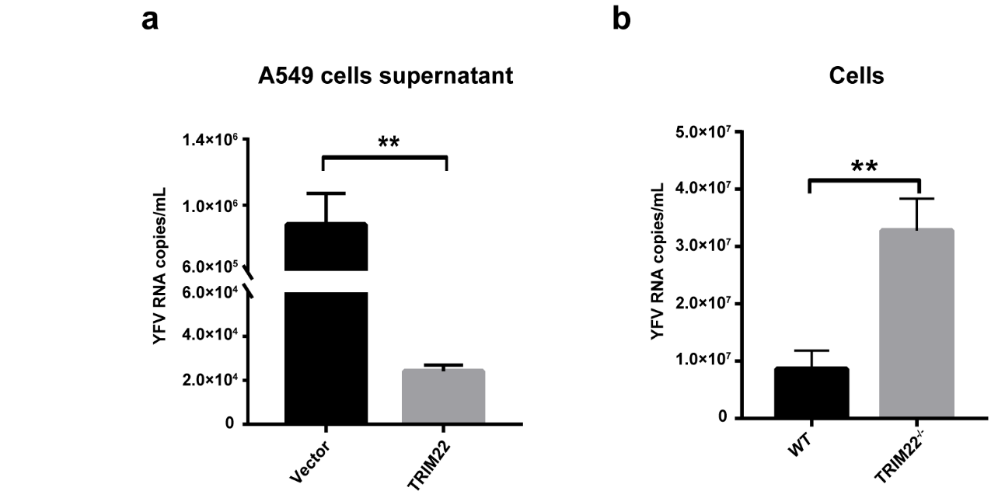


**Table S1. The list of ISG, ZIKV, DENV and YFV primers used in this study**
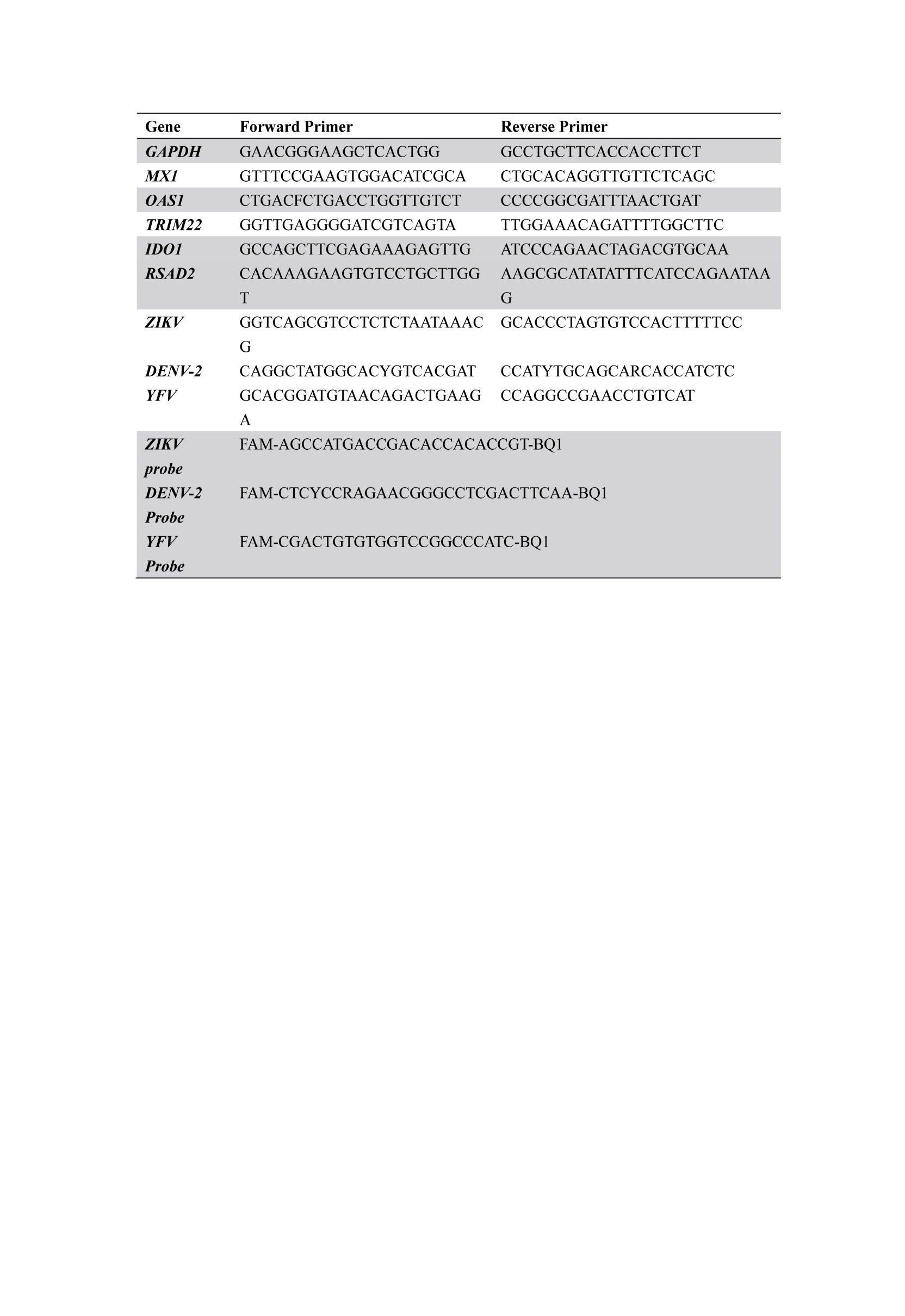

Supplement: Supplementary file 1 — Additional file 1: Figure S1. ISGs are induced by ZIKV infection in A549 cells. a-c qRT-PCR analysis of MX1, IDO1 and RSAD2 mRNA in A549 cells stimulated with increasing dose of ZIKV (FSS13025) infection. Figure S2. TRIM22 inhibits ZIKV infection in vitro. a Vero cells in 24-well plate were transfected with increasing amount of pM01-TRIM22 plasmid, 12 h later, the cells were infected with ZIKV GZ01 at MOI = 0.01, IFA of ZIKV E protein was conducted at 24 hpi. Scale bar, 100 μm. b Western blot analysis of lysates from A549 cells and TRIM22−/− A549 cells. c-d SH-SY5Y cells were transfected with pM01-TRIM22 plasmid, 12 h later, the cells were infected with ZIKV GZ01 at MOI = 0.1, ZIKV loads in cell lysates were measured by qRT-PCR at 24 hpi (c) and 48 hpi (d). e Western blot analysis of lysates from SH-SY5Y cells which were transfected with either NC siRNA or TRIM22 siRNAs. f SH-SY5Y cells were transfected with NC siRNA or TIRM22 siRNAs, 24 h later, the cells were infected with ZIKV GZ01 at MOI = 0.01, ZIKV loads in cell lysates were measured by qRT-PCR at 48 hpi. Figure S3. Model of BiLC system and IFA used to test the interactions between TRIM22 and ZIKV proteins. a Work model of GlucN-TRIM22 and GlucC-ZIKV NS1 or NS3 in BiLC system. b IFA of A549 cells that were co-transfected with pHA-TRIM22 and pHis-NS1 or pHis-NS3 plasmids, stained with HA and His tag antibody, then imaged by fluorescence microscope. Scale bar, 10 μm. Figure S4. TRIM22 mediates the K48 ubiquitylation of ZIKV NS1 and NS3 proteins. Western blot analysis of total cell lysates or immunoprecipitated proteins from HEK293T cells co-transfected with pM01-TRIM22, pHis-NS1 or pHis-NS3 and pHA-K48 (a) or pHA-K63(b) plasmids and treated with MG132, as indicated. Figure S5. TRIM22 inhibits YFV infection in vitro. a TRIM22 was over expressed in A549 cells, the cells were subsequently infected with YFV at MOI = 0.01, 12 h post transfection, YFV load in culture supernatant was measured by qRT-PCR. b TRIM22−/−A54 [file 13578_2022_872_MOESM1_ESM.docx]
